# Rectus Abdominis Endometrioma after Caesarean Section

**DOI:** 10.1155/2016/4312753

**Published:** 2016-11-23

**Authors:** Igor Mishin, Anna Mishina, Sergiu Zaharia, Gheorghe Zastavnitsky

**Affiliations:** ^1^First Department of Surgery “N. Anestiadi” and Laboratory of Hepato-Pancreato-Biliary Surgery, Medical University “N. Testemiţanu”, Chișinău, Moldova; ^2^Institute of Emergency Medicine, Chișinău, Moldova; ^3^Department of Gynecologic Surgery, Institute of Mother and Child, Chișinău, Moldova

## Abstract

Isolated rectus abdominis muscle endometriosis is very uncommon with less than 20 case reports being published to date since its first description in 1984 by Amato and Levitt. We report another case of isolated rectus abdominis endometriosis in a 37-year-old patient with a previous caesarian section. We also discuss the diagnostic and treatment particularities in these patients. In our case, the treatment was only surgical and currently the patient is disease-free during the 24-month follow-up.

## 1. Introduction

Endometriosis is defined as a functional ectopic endometrial tissue. Anterior abdominal wall endometriosis is defined as functional extraperitoneal endometrium. Endometriosis is not unusual in reproductive age patients and can be located in almost any organ. The most common affected sites are ovaries, sacrouterine ligament, rectovaginal septum, and pelvic peritoneum [1]. Less common affected sites are vulva, vagina, appendix, stomach, liver, chest, bladder, umbilicus, and inguinal canal [1]. Isolated rectus abdominis muscle endometriosis is very uncommon with less than 20 case reports being published to date since its first description in 1984 by Amato and Levitt and the best majority of them are associated with previous surgical procedures [2]. The pathophysiology of endometriosis is quite obscure with several theories being advocated to date: the implantation or reflux theory, the direct extension theory, the coelomic metaplasia theory, the induction theory, the embryonic rest theory, the lymphatic and vascular metastasis theory, and the composite theory combining implantation and vascular and lymphatic metastasis [3]. The extrapelvic (including rectus abdominis muscle) endometriosis is best explained by iatrogenic transplantation of the endometrium during a surgical procedure, as well as endometrial lymphatic and vascular migration [3, 4]. Regardless of the recent advances in imaging techniques, the preoperative diagnosis is difficult and is often confirmed after excised sample histological examination.

All medical treatments for endometriosis are equally effective in relieving pain. However, all of them alleviate pain symptoms for as long as they are used, but pain always relapses when medication is discontinued. Therefore, medications need to be used in the long term [5]. The only effective and safe treatment modality is the surgical removal of the endometrioma, especially in the context of potential malignant transformation of the lesion [6].

We report an additional case of this very uncommon condition as well as the literature review.

## 2. Case Report

**Figure 1:**
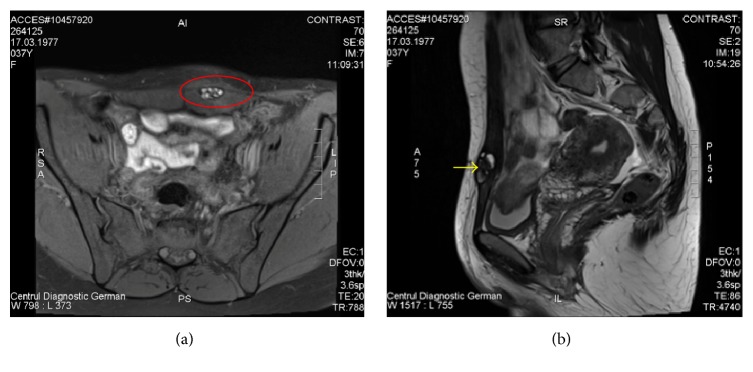
MRI: 21 × 23 × 28 mm heterogeneous mass in the lower left rectus abdominis muscle with focal areas of high and low signal intensity.

A 37-year-old patient presented due to painful mass in the left hypogastric area, in the site of a Pfannenstiel incision scar as a result of a caesarian section 3 years previously. Her past medical history was otherwise insignificant. The pain intensity and the mass size seemed to increase during menses. Upon physical examination, a firm pseudotumorous mass in the left side of the postoperative scar of about 2 cm in diameter was palpated. Her level of CA 125 (blood sample collected during the first day of menstrual cycle) was within normal limits, 25 units/mL (reference range: 0–35 kU/L). Due to the cyclic pain related to menses and tumorous mass in the site of a C-section scar, anterior abdominal wall endometriosis was suspected and the patient was scheduled for ultrasound and magnetic resonance imaging (MRI). Ultrasound with Doppler revealed hypoechoic, heterogeneous roundish nodule in the left end of the postoperative scar, with single vascular pedicle entering at the periphery of the mass. The MRI exam evidenced a 21 × 23 × 28 mm heterogeneous mass in the lower left rectus abdominis muscle with focal areas of high and low signal intensity, suggesting old haemorrhage or fibrosis (Figures 1(a) and 1(b)). The MRI scan evidenced no abdominal or pelvic cavity involvement. Elective surgery was performed for presumptive anterior abdominal wall endometrioma.

**Figure 2:**
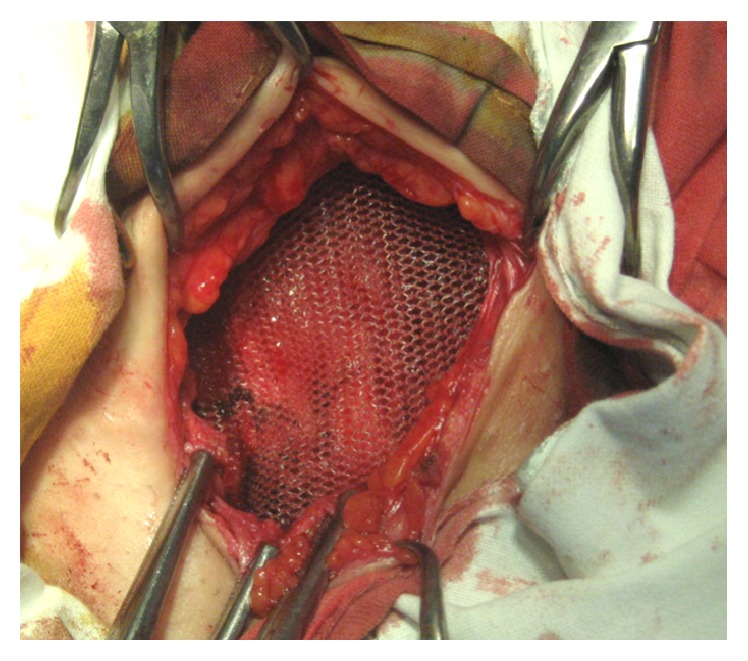
Anterior abdominal tension-free procedure using polypropylene mesh following R0 resection of rectus abdominis endometrioma.

**Figure 3:**
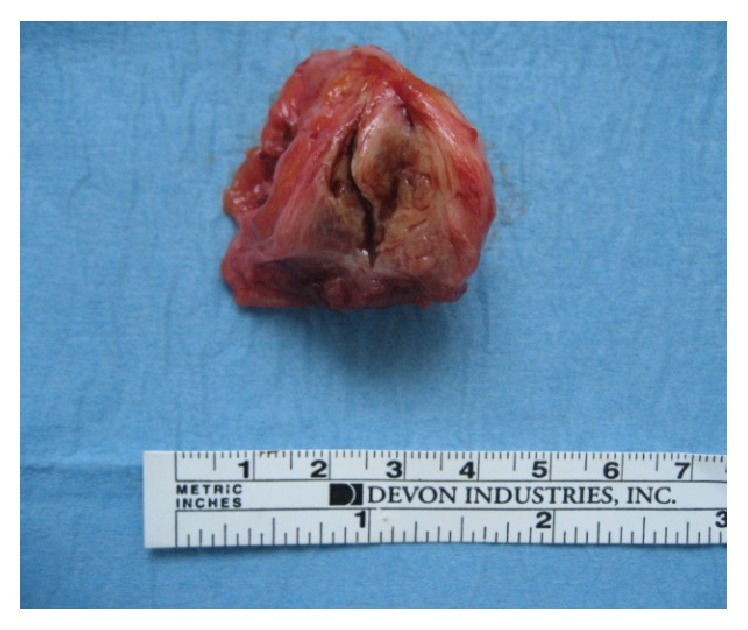
Removed surgical specimen of rectus abdominis endometrioma.

**Figure 4:**
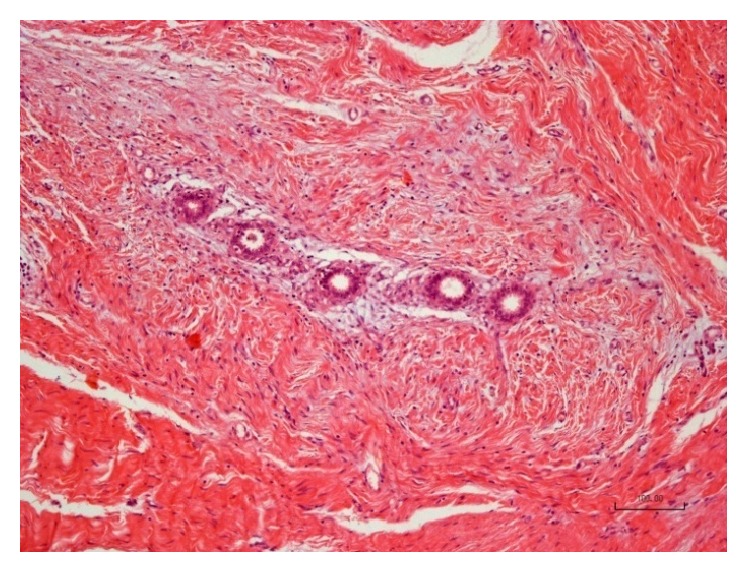
Endometrial glands and stroma (H&E, ×10).

**Figure 5:**
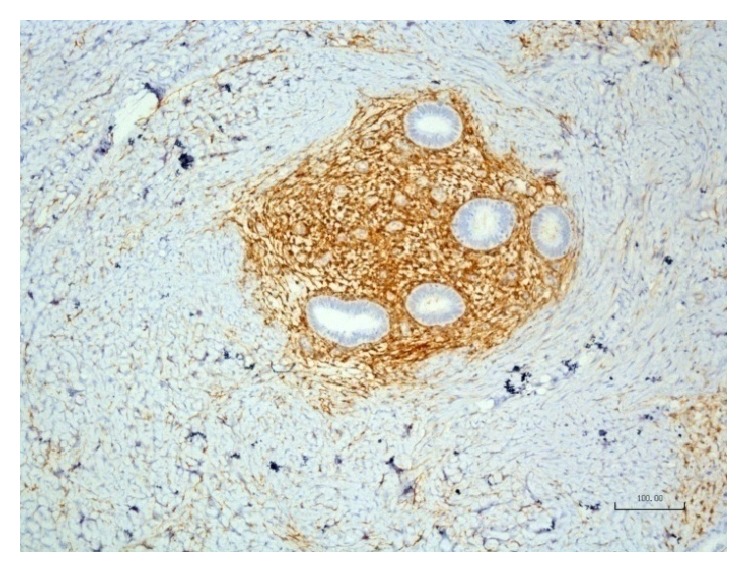
Positive immunohistochemically stained CD 10 endometrial stroma (DAB ×10).

During surgery, straight below the aponeurosis of the rectus abdominis muscle, a dense tumorous mass was observed and its complete excision including a 7–10 mm safety area was performed. Hemostasis was achieved by ligation of the vascular pedicle entering at the periphery of the mass as well as by electric coagulation. A single dose of ceftriaxone (2 g) was given intraoperatively. Due to a significant defect in the aponeurosis, it was decided to close it in a tension-free procedure, so a polypropylene mesh was used (Figure 2). The removed specimen (Figure 3) was sent for histology and immunohistochemistry examination, which confirmed endometrial glands and stroma (Figure 4) and positive immunohistochemical CD 10 endometrial stroma staining (Figure 5). The postoperative evolution was uneventful, and the wound healed* per primam. *The 24-month follow-up showed no disease recurrence.

Since there was R0 resection and her CA 125 was within normal limits without signs of malignancy upon histology examination, no gynecological oncologist was involved in the follow-up of this patient.

## 3. Discussion

Endometriosis is considered a common gynecological pathology and its frequency reaches up to 15% [7]. The most affected structures are the ovaries (up to 80%) [1]. Less common affected sites are sacrouterine ligament, rectovaginal septum, pelvic peritoneum, Douglas space, anterior rectosigmoid region, vulva, vagina, appendix, stomach, liver, chest, bladder, umbilicus, inguinal canal, pleura, and lungs, although any organ can be affected [1].

To date, there are several theories to explain endometriosis: the implantation or reflux theory, the direct extension theory, the coelomic metaplasia theory, the induction theory, the embryonic rest theory, the lymphatic and vascular metastasis theory, and the composite theory combining implantation and vascular and lymphatic metastasis [3]. The intraperitoneal endometriosis could be explained by retrograde menses.

Anterior abdominal wall endometriosis accounts for up to 1% of all endometriosis localization cases described in the literature [8]. It is considered to be a result of iatrogenic implantation usually during a gynecological procedure (most frequent caesarean section) [1], although in the literature there are described cases of anterior abdominal wall endometriosis without any history of previous surgical procedures [9–11].

The diagnosis is challenging since the clinical signs are not specific and the differential diagnosis includes suture granuloma, lymphadenopathy, abscess, inguinal hernia, incisional hernia, primary or metastatic cancer, lymphoma, lipoma, hematoma, sarcoma, desmoids tumor, and subcutaneous and sebaceous cysts [3]. Still, endometrioma of the rectus abdominis wall must be suspected in the context of a female patient of reproductive age presenting with anterior abdominal wall tumorous mass and cyclic pain depending on menses, as well as a previous history of gynecological surgery (more common caesarean section) [1], or even laparoscopic procedures [12]. It is logical to assume that, with the advance of laparoscopic surgery, including endometriosis, port site endometriosis will be more frequently reported in the future.

Recent advances in imaging techniques (USG, MRI, and CT) made the diagnosis of abdominal wall endometriosis easier, although the imaging signs are not specific and are not very informative outside of the clinical context (female patient of reproductive age presenting with anterior abdominal wall tumorous mass and cyclic pain depending on menses, as well as a previous history of gynecological surgery) [13].

Another diagnostic procedure, fine needle aspiration (FNA), was reported to be useful as well for excluding malignancy, but it seems to be inconclusive in up to 75% of cases. Moreover, FNA is associated with an increased recurrence risk [14]. Under these circumstances, we did not consider this diagnostic procedure in our patient.

The treatment of choice for endometriosis of the rectus abdominis muscle is a wide local excision of the lesion with negative margins. The surgical excision should include 5–10 mm of the surrounding healthy tissue as surgical margin and care must be taken not to rupture the mass to avoid reimplantation of microscopic remnants of endometrial tissue [15]. The same procedure was followed in our case.

To date, medical therapy for abdominal wall endometrioma is considered updated since it is unsuccessful and permits just temporary symptoms relief [7]. Medication using progestins is considered safe, effective, and well tolerated and some authors advocate it as first-line treatment in symptomatic patients with endometriosis who do not want to have children [5], but this was not the case in our patient. All medical treatments for endometriosis are equally effective in relieving pain. However, all of them alleviate clinical signs just for as long as they are used, but still pain always relapses once the medication is discontinued. Therefore, medications need to be used in the long term. Thus, the only effective and safe treatment modality is the surgical removal of the endometrioma, especially in the context of potential malignant transformation of the lesion [6].

Recurrence is rare, usually presents within 1 year, and is likely to be the result of an inadequate excision [8]. To date, our patient has been disease-free for 24 months.

## 4. Conclusion

We presented a rare case of anterior rectus abdominis muscle endometriosis diagnosed preoperatively and confirmed by histopathology and immunohistochemistry CD 10. The treatment of this exceptional condition should be surgical and this clinical entity must be included in the differential diagnosis of any abdominal mass in fertile female patients with or without surgical history.
